# Fuzzy Logic for Intelligent Control System Using Soft Computing Applications

**DOI:** 10.3390/s21082617

**Published:** 2021-04-08

**Authors:** Catalin Dumitrescu, Petrica Ciotirnae, Constantin Vizitiu

**Affiliations:** 1 Department Telematics and Electronics for Transports, University “Politehnica” of Bucharest, 060042 Bucharest, Romania; 2 Communications Department of Military Technical Academy “Ferdinand I”, 39-49 George Cosbuc Avenue, 050141 Bucharest, Romania; petrica.ciotirnae@mta.ro (P.C.); constantin.vizitiu@mta.ro (C.V.)

**Keywords:** fuzzy logic control, path planning, fuzzy interference system

## Abstract

When considering the concept of distributed intelligent control, three types of components can be defined: (i) fuzzy sensors which provide a representation of measurements as fuzzy subsets, (ii) fuzzy actuators which can operate in the real world based on the fuzzy subsets they receive, and, (iii) the fuzzy components of the inference. As a result, these elements generate new fuzzy subsets from the fuzzy elements that were previously used. The purpose of this article is to define the elements of an interoperable technology Fuzzy Applied Cell Control-soft computing language for the development of fuzzy components with distributed intelligence implemented on the DSP target. The cells in the network are configured using the operations of symbolic fusion, symbolic inference and fuzzy–real symbolic transformation, which are based on the concepts of fuzzy meaning and fuzzy description. The two applications presented in the article, Agent-based modeling and fuzzy logic for simulating pedestrian crowds in panic decision-making situations and Fuzzy controller for mobile robot, are both timely. The increasing occurrence of panic moments during mass events prompted the investigation of the impact of panic on crowd dynamics and the simulation of pedestrian flows in panic situations. Based on the research presented in the article, we propose a Fuzzy controller-based system for determining pedestrian flows and calculating the shortest evacuation distance in panic situations. Fuzzy logic, one of the representation techniques in artificial intelligence, is a well-known method in soft computing that allows the treatment of strong constraints caused by the inaccuracy of the data obtained from the robot’s sensors. Based on this motivation, the second application proposed in the article creates an intelligent control technique based on Fuzzy Logic Control (FLC), a feature of intelligent control systems that can be used as an alternative to traditional control techniques for mobile robots. This method allows you to simulate the experience of a human expert. The benefits of using a network of fuzzy components are not limited to those provided distributed systems. Fuzzy cells are simple to configure while also providing high-level functions such as mergers and decision-making processes.

## 1. Introduction

The rapid growth of interest in the development of intelligent systems has led to the multiplication of sensor types, which provide an increasingly rich perception of the surrounding reality, allowing the description and solving of various problems. The use of new types of sensors and actuators leads to better quality, productivity, and process safety. The general trend is a rapid increase in the number of measurements and actions in the control of complex processes. Therefore, the needed processing power must increase accordingly. Currently, no single microcontroller/processor is used to simultaneously perform the measurements, the decision, and the process control loop, thus switching to distributed intelligent processing. This intelligent processing is done through a spatial distribution, using certain logic, so that the cooperative solutions between the distributed processors can be easily identified. The distribution of components should, as far as possible, ensure decentralization of computing capabilities without substantially reducing the overall reliability of the system. Communication processes between functional components should be limited; otherwise, the performance could decrease dramatically. The use of communication systems ensures the exchange of data between distributed processors, thus achieving the first stage of intelligence.

In addition, intelligent components must be able to process the data addressed to them locally. This second level of intelligence is needed to reduce the exchange of information as smart tools produce increasingly complex data. This new approach will allow a faster exchange of data between system components and, at the same time, will ensure the uniform and compact treatment of informational data coming from different sources. Thus, distributed systems will perform complex functions of managing the entire distributed network. These functions will be referred to as interoperability and interfunctional coordination. To achieve the concepts described above, it is necessary to access means that allow the integration of intelligence at lower levels. Due to the ability to represent gradual information in a way familiar to human thinking, fuzzy logic is a powerful tool for integrating intelligence. In particular, the connection between linguistic terms and numerical quantities allows the implementation of high-level functions, such as data fusion and complex decision-making processes. The structure of fuzzy circuits is generally characterized by the number and shape of input and output variables, the number of rules it evaluates simultaneously, the type of inferences and defuzzification methods [1]. The performance of fuzzy circuits will be evaluated according to the speed of data processing (number of fuzzy inferences per second-FLIPS), the accuracy of the results (errors, internal noise of analog circuits and the number of bits needed to represent the values of fuzzy calculations) [2,3]. A fast response of circuits that implement nonlinear functions such as MIN and MAX, whose output signals may be subject to discontinuities, is required [4,5]. Mamdani processors are currently applied in process control, robotics, and other expert systems [6]. They are particularly suitable for the execution of command-and-control actions of an operator. They lead to good results that are often close to those of a human operator, eliminating the risk of human error [7,8,9]. Takagi-Sugeno processors are mainly used in process modeling [9,10]. Fuzzy processors are made by observing the control activity of the operators as the modeling process [11], thus acquiring a large amount of data while the operator executes the control [12,13].

The Sugeno processor is a particular case of the Takagi-Sugeno processor [12]. The particularity of distributed systems is that all output functions are precise values, and the consequence functions will be replaced by constants that are weighted by the “decisions” of truth established for the initial conditions to obtain the final output results. The fuzzy sets that form these exact values are generally called “singletons” or Sugeno sets [14,15].

Fuzzy logic, one of the techniques of representation in artificial intelligence, is a well-known method in soft computing that allows the treatment of strong constraints generated by the inaccuracy that characterizes the data obtained from sensors. Fuzzy control is an intelligent control technique, characteristic of intelligent control systems, which serves as an alternative to conventional control techniques, and the construction of a mathematical model is not necessary. The article presents two intelligent control applications, Agent-based modeling and fuzzy logic for simulating pedestrian crowds in panic decision-making and Fuzzy controller for mobile robot.

The increasing occurrence of moments of panic during mass events motivated the study of the impact of panic on crowd dynamics and the simulation of pedestrian flows in panic situations. The lack of understanding of moments of panic continues to cause hundreds of casualties each year, not to mention methodological studies of panic behavior capable of predicting crowd dynamics. Under these conditions, there are thousands of casualties and twice as many accidents caused by the crowd around the world, despite efforts to control the crowd and a large number of security forces. Based on this simulation, a system was developed for determining pedestrian flows and calculating the optimal evacuation distance in panic situations. The system is based on the creation of a network of sensors for receiving Bluetooth signals from mobile devices and the use of fuzzy logic to determine the position of pedestrians in relation to escape routes by calculating the optimal escape distance. This system can be used inside buildings in panic situations that require the evacuation of the population, but also for anonymous monitoring of a person’s route inside clinics and shops. The system can also be applied to open spaces for monitoring the urban mobility of pedestrians and vehicles, with applicability for Crisis Management systems (e.g., mobility of people during the COVID pandemic). We created a new intelligent control interface for the evacuation of people in panic situations in order to improve the performance of evacuation mobility from closed/open spaces, a useful application in operations to save lives, in crisis situations, earthquakes, fires or terrorist actions-CBRNE.

Fuzzy Logic Control (FLC) is suitable for controlling a mobile robot because it can interfere even when the data acquired by the robot’s sensors is inaccurate. Based on this, we built a Fuzzy controller for mobile robots that uses Bezier curves to evaluate the trajectory and monitor the robot’s movement. This approach offers the opportunity to simulate the experience of a human expert. However, the lack of systematic learning capacity in the design of fuzzy logic-based systems has sparked a particular interest in combining fuzzy logic with other special learning methods, such as neural networks, in order to achieve flexible behavior. Flexible behavior involves the ability to learn-to acquire knowledge or to improve skills, based on experience, observations, or training.

The article is organized as follows: first, we present a brief overview of the mathematical concept for fuzzy control; then, we describe the language Fuzzy Applied Cell Control Technology-soft computing, and finally, we present two applications for Intelligent Systems and Control

## 2. Related Work

Currently, the planning of routes for the movement of robots plays an important role in their mobility to avoid obstacles. Consequently, the development of smart and autonomous systems refers to the academic and industrial environments in the R&D segment, given the improved efficiency of smart systems. Intelligent systems use artificial intelligence and pattern recognition algorithms to detect events, make decisions, and ultimately achieve the most complex control of systems. Currently, developments for robot control using fuzzy-based intelligent obstacle-avoidance strategy have been reported in the literature [16,17,18]. Fuzzy algorithms for the design, modeling and implementation of a fuzzy controller have been presented in the literature for an intelligent overtaking system using neuro-fuzzy controllers [19] and the design of a robust adaptive fuzzy controller for a single input–single class (SISO) Uncertain nonlinear systems [20]. Moreover, other authors introduced a real-time optimal path planning of humanoid robots [21,22,23], and Hongtao X and the team presented a fuzzy algorithm for controlling the direction of a robot [24]. The authors [25] presented a control architecture for automatic direction preservation using PID algorithms with fuzzy logic.

Researchers [26,27] proposed trajectory tracking control of a four-wheeled omnidirectional mobile robot based on the reference model approach.

Other authors have presented algorithms for steering control [28,29]. For localization, using different fuzzy controllers and localization algorithms has been proposed in [30,31,32,33]. The applications proposed by us in this article are in a way of a novelty character, especially fuzzy control applications for estimating the dynamics of the pulpation in panic conditions and designing a Mamdani type fuzyy controller with two outputs.

As a result, fuzzy logic can be roughly equated to Computational Word (CW). CW is a methodology that differs from the traditional definition of computing, which is the manipulation of numbers and symbols. As a direct consequence, CW provides a methodology for bridging the gap between human brain mechanisms and machine processes to solve problems by equipping computers with tools to deal with imprecision, uncertainty, and partial truth. CW [34] deals with words and propositions from a natural language as the main objects of computation, for example: “small”, “big”, “expensive”, “quite possible” or even more complex sentences as “tomorrow will be cloudy but not very cold”. The main inspiration of CW is the human ability of performing several different tasks (walk on the street, play football, ride a bicycle, understand a conversation, making a decision) without needing an explicit use of any measurements nor computations. This capability is sustained by the brain’s ability to manipulate different perceptions (usually imprecise, uncertain, or partial perceptions), which plays a key role in human recognition, decision, and execution processes. In recent years, many researchers have seen CW as a very interesting methodology to be applied in decision making [35]. As it allows to model perceptions and preferences in a more human-like style and it can provide computers some of the needed tools, if not to fully simulate human decision making, to develop complex decision support systems to ease the decision makers to reach a solution [36].

## 3. Materials and Methods

In this article, we will not present the theory of fuzzy logic in detail, because we did this in a previous article “Aircraft Trajectory Tracking Using Radar Equipment with Fuzzy Logic Algorithm*”*, where in Sections 2.1–2.3, we detailed these elements [37]. In this article, we will focus on how to achieve an optimal fuzzy controller and on the implementation of the architecture and functionalities of fuzzy processors.

Fuzzy processors are programmable like standard microprocessors, but the execution of fuzzy operations is much faster. PC’s operation as well as the availability of a wide range of data acquisition modules makes the implementation of control systems with the help of PC’s in various programming systems/platforms to know a strong development. The integration of fuzzy logic on such platforms programming has become easy due to the use soft computing tools.

### 3.1. Fuzzy Controllers’ Processor

**Figure 1:**
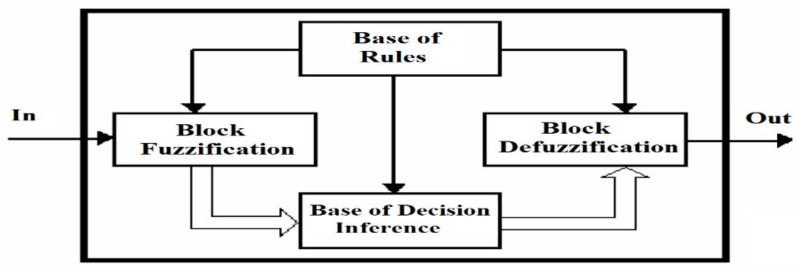
The informational structure of a fuzzy controller.

In general, a fuzzy controller has the basic structure shown in Figure 1, where the following component blocks are highlighted:

The fuzzy rules (knowledge base);

The fuzzification block;

The inference decision (inference decision);

The defuzzification block.

The fuzzification block represents the input block in the fuzzy controller, with the role of obtaining fuzzy information in the form of linguistic variables, linguistic terms and membership functions from a crisp value. This fuzzy information (fuzzy values) will be compared with the premises of all “if, then” type rules contained in the rules base and used by the inference mechanism for their activation and application.

The basis of continuous rules is the transposition into fuzzy logic of the linguistic description of the way in which an efficient control would be achieved.

Therefore, this block consists of using the set of “if” and “then” rules set by the expert and defined on the fuzzy input and output variables.

The inference mechanism expresses the way in which the rules established by the expert for the input variables are interpreted and applied. This mechanism evaluates which of the rules are relevant at the appropriate time based on the degrees of membership and decides the (fuzzy) value of the output quantity from the controller using operators appropriate to vague logic.

The defuzzification block ensures that the result obtained from the decision block, a fuzzy value, is converted into a real physical value that will be transmitted to the process/execution element. Practically, the reverse fuzzification operation will be performed here.

### 3.2. Classification of Fuzzy Processor

Fuzzy processors can perform three essential operations: real–fuzzy transformation (TRF), inference, and fuzzy–real transformation (TFR). If *X* and *L*(*X*) are the finite set of numeric values and the set of linguistic terms associated with the processor inputs, *U* and *L*(*U)* are the finite set of numeric values and the set of linguistic terms associated with the processor outputs. In this hypothesis, it is *E* any set, and *F*(*E*) is the set of fuzzy subsets of *E*. If so, *E* will represent the “discourse universe”. TRF can be represented by a function, denoted *φ*, on the interval determined by the set *X* to the fuzzy subsets associated with the processor input, which we denote by *F*(*Y*). The inference will produce a new fuzzy subset starting from the TRF result, using a lot of rules. This can be represented by a function, denoted *g*, from the set *F*(*Y*) associated with the processor input, to a set of fuzzy subsets associated with the output denoted *F*(*Z*).

**Figure 2:**
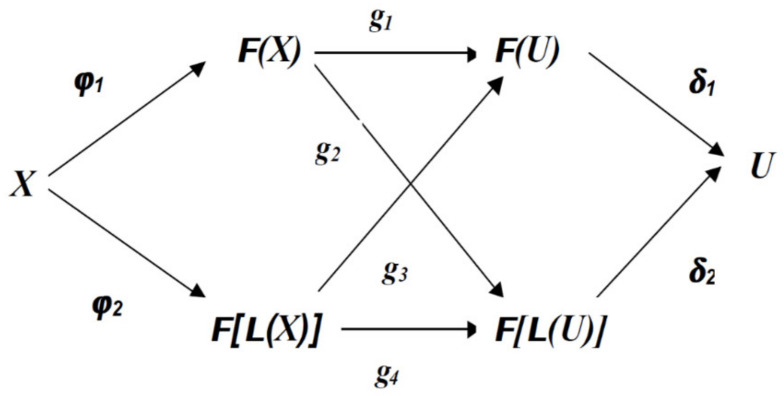
Classification of fuzzy processors into four classes.

TFR produces a real output using the inference result. The result obtained can be represented by a function, which we will denote *δ*, for the set *F*(*Z)* associated with the output, at *U*. The set *Y* associated TRF and the inference can correspond to the set of numerical values, *X*, or the set of linguistic terms, *L*(*X).* Similarly, the set *Z* associated with the inference and TFR processes can represent a set of numerical values, *U*, or a set of linguistic terms, *L*(*U).* Thus, the definitions mentioned above determine a classification of fuzzy processors into four classes, as shown in Figure 2. A fuzzy processor that uses inference *g_i_* will be called type *i* or abbreviated fuzzy processor, *CF_i_.*

### 3.3. Fuzzy Component

We can define the following components:

- Fuzzy sensors, which ensure the representation of measurements as fuzzy subsets.
- Fuzzy actuators, which operate in the real world, are depended on the fuzzy subsets they receive at the input.
- Inference fuzzy components that can perform fuzzy calculations. They produce new fuzzy subsets obtained from the received fuzzy subsets.

In general, we use two types of components: components that work with subsets of numbers and components that work with subsets of symbols. The first components can be called numeric fuzzy components and the other symbolic fuzzy components. In the mentioned classification, the fuzzy sensors are represented by the function *φ*, the fuzzy actuators by the function *δ* and the fuzzy inference components by the function *g*. Their purpose is to interconnect all the fuzzy components in a network, to reduce the amount of information they will exchange with each other. Due to the use of a small number of linguistic terms in applications, it is useful to develop symbolic fuzzy components. In the following, we will consider only the obscure symbolic components, omit the notion of “symbolic” and use only the term “fuzzy component”. The fuzzy sensors are represented by the *φ*_2_ function, the fuzzy actuators by the *δ*_2_ function and the inference components by the *g*_4_ function.

Their purpose is to interconnect all the fuzzy components in a network, to reduce the amount of information they will exchange with each other. Due to the use of a small number of linguistic terms in applications, it is useful to develop symbolic fuzzy components. In the following, we will consider only the obscure symbolic components, omit the notion of “symbolic” and use only the term “fuzzy component”. Most fuzzy control applications that rely on computers or microprocessors are programmed to make numerical inferences by sequential calculations is limited to solving problems that do not require high response speeds. Real-time systems require short response times in most cases. In this case, the hardware implementation of fuzzy systems is the only affordable solution. The digital (numerical) hardware approach of fuzzy systems will contain the logic circuits needed to execute fuzzy algorithm, memory for storing fuzzy rules and tables correspondence (look-up tables) for storing the membership functions of the input and output variables. Thus, compared to an analog approach, the digital approach offers greater flexibility in design and good compatibility with other digital systems. On the other hand, in order to communicate with sensors and actuators, most digital systems require analog-to-digital and digital-to-analog converters.

### 3.4. Fuzzy Cells

**Figure 3:**
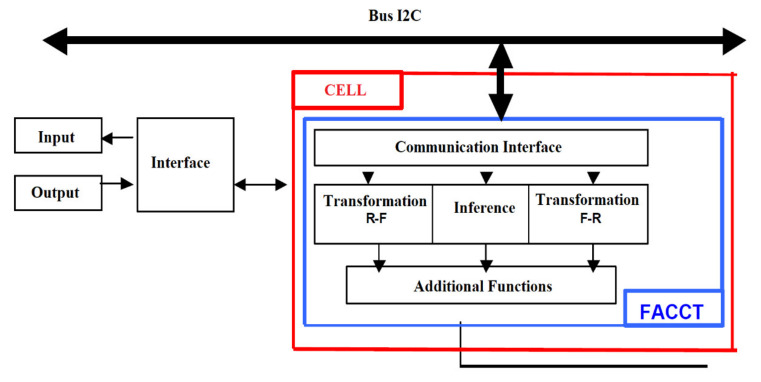
Fuzzy cell architecture.

Fuzzy components can be considered as independent applications belonging to a general model, called the fuzzy cell (Figure 3). Fuzzy cells can be implemented with the microcontroller, DSP, and FPGA. Local communications can be provided by a local bus architecture and the communication performance. The input interface can be provided by an analog converter-numerically, with which the microcontroller can be equipped. The same converter can be accessed successively by several analog channels. If the microcontroller is equipped with inputs for pulse modulation, an output interface may also be available local analog.

**Figure 4:**
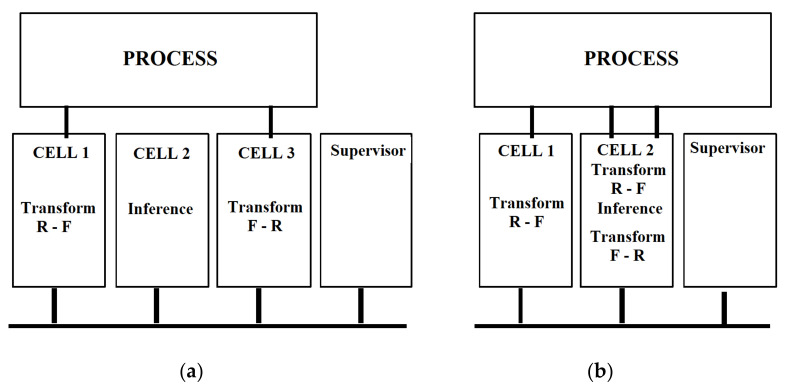
The configuration of a fuzzy cell: (**a**) configuration with a fuzzy sensor, a fuzzy actuator or an inference component; (**b**) implementations of a fuzzy sensor in cell 1 and a fuzzy controller in cell 2.

Communication between fuzzy cells can be provided on an I2C bus, which is known to have an extremely low implementation cost. However, any other communications interface supported by the microcontroller can be used. Each component connected to the I2C bus has an address that identifies it. Specialized circuits have been developed to facilitate connection to the bus I2C. Depending on the configuration we use, the fuzzy cell can be a fuzzy sensor, a fuzzy actuator, or an inference component (Figure 4a). A fuzzy sensor can be implemented in the same cells together with the inference component.

Using this principle, a fuzzy processor can be easily implemented using a single fuzzy cell. In this case, the fuzzy cell performs TRF, inference, and TFR. Figure 4b shows the implementation of a fuzzy sensor in cell 1, and in cell 2 a fuzzy controller is implemented.

### 3.5. Mathematical Models for Fuzzy Components

#### 3.5.1. Real–Fuzzy Symbolic Transformation

The symbolic fuzzification used in fuzzy cells is based on the definitions of the notions of meaning and description. If *X* is a discourse universe and *L*(*X*) a lot of linguistic terms associated with it, then the relationship between *X* and *L*(*X*) defines a fuzzy language and can be characterized by a fuzzy graph whose realization function is *µ_R_*(*x, L*)*,* where x∈X and $L\in L\left(X\right)$ for $\mu_{R}\left(x,L\right):XxL\left(X\right)\to \left[0,1\right]$. The linguistic term fuzzy is defined by the function *M*: $M:L\left(X\right)\to F\left(X\right)$ for which

(1) $$\forall x\in X,\;\mu_{M\left(L\right)}\left(x\right)=\mu_{R}\left(x,L\right)$$

The membership function $\mu_{M\left(L\right)}\left(x\right)$ shows to what extent an element of *X* can be associated with the meaning of a linguistic term.

The fuzzy description of an element of the set *X* (this is in this case a measurement) defined by the function $D:X\to F\left[L\left(X\right)\right]$, so that

(2) $$\forall L\in L\left(X\right),\;\mu_{D\left(x\right)}\left(L\right)=\mu_{R}\left(x,L\right)$$

The membership function $\mu_{D\left(x\right)}\left(L\right)$ shows to what extent the linguistic term *L* satisfies the description of an element in *X*.

**Figure 5:**
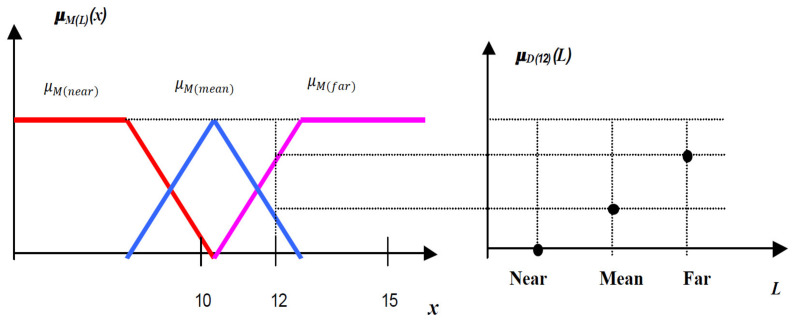
The fuzzy meanings and the fuzzy linguistic for *x* = 12.

It follows that the meaning of fuzzy and the description of fuzzy are linked by an equal relation (see Figure 5), which shows that an element is described by a linguistic term if this element also belongs to the meaning of the term

(3) $$\mu_{D\left(x\right)}\left(L\right)=\mu_{M\left(L\right)}\left(x\right)$$

Most of the time, the notation *M*(*L*) is abbreviated to *L*. Thus, instead of writing $\mu_{M\left(L\right)}\left(x\right),$ we write $\mu_{L}\left(x\right)$. It is considered that, in most cases, the context will make the difference between the term and its meaning. In this situation, the clarification is established by the precise definition of the fuzzy description whose result is a fuzzy subset defined on a set of linguistic terms. Thus, the fuzzy description becomes consistent with the symbolic fusing defined by the function *j*_2_. It turns out that the solution for implementing fuzzy sensors is to calculate the fuzzy descriptors associated with a measurement simultaneously with the definition of all fuzzy signification for all linguistic terms.

#### 3.5.2. Symbolic Fuzzy Inference

Fuzzy cells use the *g*_4_ inference, for which the relation associated with the set of rules can be defined by the Cartesian multiplication of the set of linguistic terms. If $L\left(\varepsilon \right),\;L\left(\Delta \varepsilon \right)$ and *L*(*U*)*,* respectively, representing the datasets for linguistic terms for error, error variation, and command term variation. Each control rule defines a relation on the cartesian multiplication $L\left(\epsilon \right)\times L\left(\Delta \epsilon \right)\times L\left(U\right)$, where $L\in \left(\epsilon \right),\;L^{\prime }\in L\left(\Delta \epsilon \right)$ and $L^{\prime \prime }\in L\left(U\right)$. This relationship can be represented on the fuzzy graph and the membership function is $\mu_{\Gamma }\left(L,L^{\prime },L^{\prime \prime }\right)$. For example, the principle “If the error is negative and the error variation is positive then the command is zero” leads to the conclusion that $\mu_{\Gamma }\left(negative,\;positive,\;zero\right)=1$.

The result of the inference results from the law of composition. With two inputs and the operator min as the triangular rate, inference is relation below (4), resulting in a fuzzy subset of *L*(*U*).

(4) $$\begin{array}{c} \forall L^{\prime \prime }\in L\left(U\right),\;\mu_{F}\left(L^{\prime \prime }\right)=\;min\left\{min\left[\mu_{\rho 2\left(\varepsilon \right)}\left(L\right),\mu_{\rho 2\left(\Delta \epsilon \right)}\left(L^{\prime }\right)\right],\mu_{\Gamma }\left(L,L^{\prime },L^{\prime \prime }\right)\right\}\;\\ L\in L_{\left(\epsilon \right)},L^{\prime }\in \left(\Delta \varepsilon \right) \end{array}$$

In this expression, *max* is the projection operator, and *L*(*U*) represents the finite set of linguistic terms. The first *min* operator represents the combination operator that performs the conjunction between the input and the representation of the fuzzy graph. The second *min* operator represents the aggregation of the inputs and determines the establishment of the rules.

#### 3.5.3. Fuzzy–Real Symbolic Transformation

TFR is one of the main processes used in fuzzy control. The classical models of TFR, represented by the $\delta_{1}$ in Figure 2, are the Mean Maximum (MoM) and the Center of Area (CoA). The Fuzzy–Real symbolic transformation, represented by the function $\delta_{2}$ in Figure 2, makes the connection between the Fuzzy subset set *F*[*L*(*U*)] and the action control, *U*. We can approach several calculation variants for the symbolic Fuzzy–Real transformation. In this article we will choose the method that considers the function $\delta_{2}$ as a unification of the other two functions mentioned above. The first, denoted $\delta_{3}:\left[L\left(U\right)\right]\to F\left(U\right),$ will transform the result of the inference, from a fuzzy subset *L*(*U*)*,* into a fuzzy subset belonging to the set *U*. The second function, denoted $\delta_{1}$, represents the classical method defuzzification.

Knowing the meanings of the symbols in *L*(*U*)*,* the relation $\delta_{3}$ is analogous in principle with an inference of type *g*_3_. It can be calculated (using the law of composition of inference) because of the fuzzy subset signification of these symbols. If the function associated with the inputs is $\mu_{F}$, it results

(5) $$\forall u\in U,\;\mu_{G}\left(u\right)=\;min\left[\mu_{F}\left(L\right),\mu_{M\left(L\right)}\left(u\right)\right]\;$$

Using the center of area method, the numerical value return by the fuzzy actuator is

(6) $$U=\delta_{2}\left(F\right)=\frac{\int \mu_{G}\left(\gamma \right).\gamma .d\gamma }{\int \mu_{G}\left(\gamma \right).d\gamma },\;\gamma \in U_{s}$$

and the discrete variant through

(7) $$\mathrm{CoA}\left(F\right)=\frac{\sum_{q=1}^{N_{q}}\mu_{B}(y_{q})y_{q}}{\sum_{q=1}^{N_{q}}\mu_{B}(y_{q})}$$

where $N_{q}$ is the number of quanta used to discretize the membership function $\mu_{B}\left(y\right)$ of the fuzzy output *B*.

**Figure 6:**
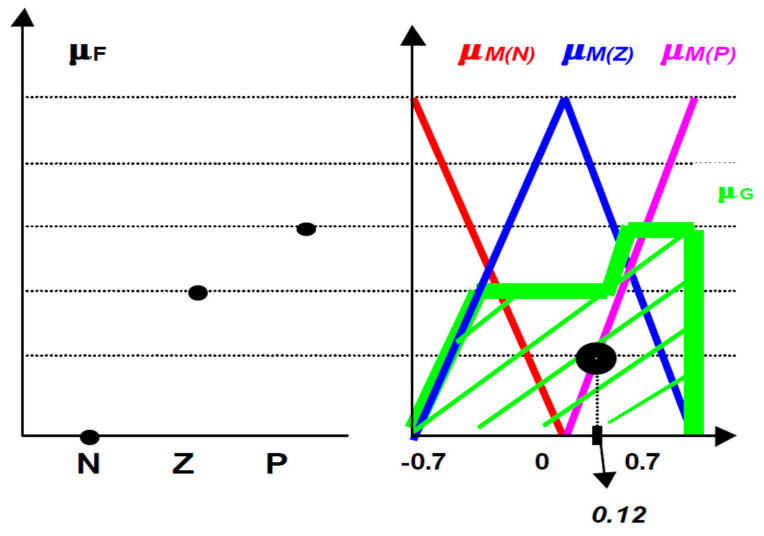
The proposed solution for defuzzification obtained with a triangular norm min which leads to the result μ=0.12.

Let fuzzy subset be $F:\left\{\frac{0}{N},\frac{0.4}{Z},\frac{0.6}{P}\right\}$. In Figure 6 is proposed a defuzzification solution obtained with a triangular norm min which leads to the result μ=0.12.

### 3.6. Fuzzy Cell Configuration

#### 3.6.1. Use a Local Compiler and Soft Computing

Setting up a fuzzy cell should be as simple as possible. Due to the distribution of fuzzy operations in several cells, the solution based on the unique system configuration must be avoided. Defining the rules and fuzzy meanings associated with linguistic terms is similar globally. However, none of them allows easy implementation of distributed systems for TRF, inference and TFR.

That is why new languages need to be developed, specially designed to configure fuzzy cells. We call such a language Fuzzy Applied Cell Control Technology (FACCT).

**Figure 7:**
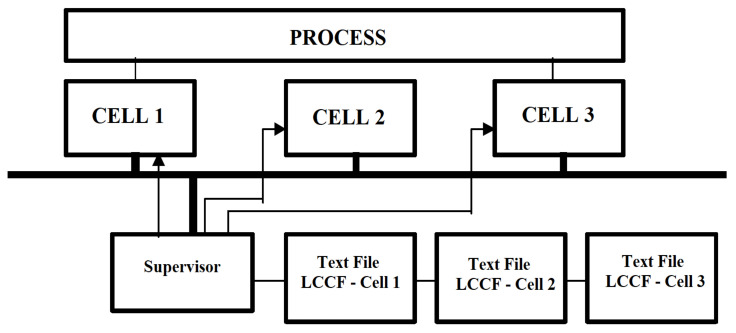
Configuration of three fuzzy cells connected to a computer that functions as a supervisor.

A FACCT compiler must be integrated into each fuzzy cell. After receiving a FACCT file, the fuzzy cell must call the compiler. It generates an internal representation of the file that is processed by the cell. FACCT files can be transmitted by any component connected to the network, these can be other fuzzy cells but also processing computers. Figure 7 shows a three-cell configuration fuzzy connected to a computer that works as a supervisor.

Inserting a compiler at the cell level is not a common practice, so some explanations are needed. The first reason would be an active configuration at any time for connecting components and easily configurable, unlike the classic solution in which the use of a microcontroller in the cell configuration leads the compilation and link-editing outside the system.

The second reason would be the advantage of soft computing distribution. It is commonly accepted that a smart component (smart sensors or smart actuators) contains local processing capabilities. Therefore, the association between the “intelligence” of a component and the use of microprocessors, DSP or FPGA is always made. In fact, intelligence is included in processor operation algorithms rather than local configuration. It is also possible admits that a component could be considered intelligent without having local processing capabilities. Suppose a rapid Fourier transformation is required in the event of an exceptional situation. The classic solution leads to the implementation of an intelligent sensor based on a microprocessor powerful enough to process such an algorithm, even if the probability of occurrence of the exception it is small. Thanks to the component interconnection bus, one can imagine components that have locally stored algorithms that can be executed on any remote resource in the network. We call these components “Distributed Intelligence Components.” The realization of this solution implies the existence of an interoperable language, so that all computing resources, regardless of the type of processor used, can perform calculations and exchange information.

**Figure 8:**
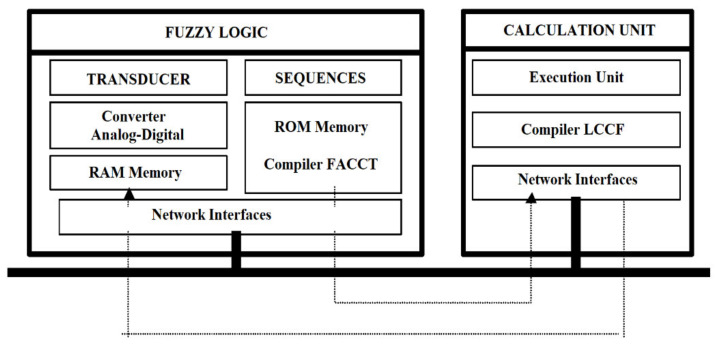
Development fuzzy components with distributed intelligence using the Fuzzy Applied Cell Control Technology (FACCT) programming language.

From this point of view, FACCT can be considered an interoperable language for the development of fuzzy components with distributed intelligence (Figure 8). Thus, fuzzy cells and fuzzy computing resources that use the FACCT language can make a resident compiler.

#### 3.6.2. Soft Computing Implementation of Fuzzy Applied Cell Control Technology

Now we understand by the Fuzzy Applied Cell Control Technology soft computing language a general method of solving a certain type of problem, which can be implemented on the computer. In this context, an algorithm is the absolute essence of a routine. The programming language that underlies the fuzzy machine includes, in addition to the logical part, an algebraic part. It is therefore a mixed type of algorithm, organized as a finite sequence of steps, comprising several specific operations. They fully meet the basic conditions to be implemented on the computer, i.e., they are defined and effective. The form that the algorithm takes in a computer implementation is subordinated to the programming style and depends especially on the type of language. The software implementation of the fuzzy automaton can also be done based on a parallel algorithm. Parallel computing gives a new dimension to the construction of Fuzzy Applied Cell Control Technology algorithms and programs. It is emphasized that parallel programming is not a simple extension of serial programming and that not all sequential algorithms can be parallelized.

In the case of Fuzzy Applied Cell Control Technology soft computing implementations, the synthesis of fuzzy automata (controllers) is sufficiently flexible, being practically a problem of emulating the typical phases of the algorithm, for the model of the given problem. The ability to program a problem is important in this case. However, some considerations are needed, which must be considered when structuring a fuzzy control system, regardless of the form in which it will be implemented. The configuration of a fuzzy controller, intended to lead a process, considers the conventional decomposition of its dynamics, corresponding to the evolution strategies adopted in the modeling stage.

From the command point of view, the fuzzy controller will be structured on control channels depending on the type of dynamic controlled system: single-input/single-output (SISO), multiple -input/single-output (MISO) and multiple-input/multiple-output (MIMO). The problem of control channel independence is analyzed in the context of the existence of the informational coupling. The interdependence of the channels is imposed by the methodology of leading the control process, taking it into account when describing the heuristic basis of the problem and in the stage of compiling the rule base.

This is important, especially in software implementation, because the way information is processed depends on the sequential operating principle and the limited possibilities to parallelize the calculations. The possibilities of direct hardware implementation of fuzzy systems are currently a reality due to the appearance of fuzzy logic circuits, elementary machines for performing fuzzy inferences and circuits for generating characteristic functions with controlled membership. These microelectronic systems are found in the structure of dedicated or general-purpose fuzzy processors.

*Designing the Fuzzy Applied Cell Control Technology Architecture*

To implement the mathematical models developed for special applications that we present in the next chapter, it is necessary to design each component of the structure of a fuzzy system: fuser, inference motor and defuzzification. The solution proposed for the implementation of the decision-making system is based on the classic structure of a fuzzy system with some specific modifications. The proposed architectures implement the fuser based on the membership functions defined in the table by means of memory blocks. The capacity of each memory block depends directly on the value range of the parameter it defines. To define the membership functions and their shapes, the relative variation of the wire resistance and the variation of the pressure in the glass tube were used. The implementation of this method of fusing involves the use of memory circuits, which offers the possibility of defining non-linear membership functions, different from the classical ones, which can be quite convenient in case of more specific problems. The main advantage of the fuzzifier implemented with this method is the possibility to render some rather complex nonlinear membership functions. Another advantage is the use of memory blocks with the help of which membership functions can be defined which can be subsequently changed dynamically. The main disadvantage of the given approach is the use of a large memory capacity to define all the membership functions of the fuzzy variable qualifiers. The memory capacity used can be reduced by decreasing the value ranges. To define the membership functions in the table and to register them in the RAM/ROM memory blocks, it was necessary to perform the procedure for adjusting them. The given procedure consists in translating the range of values to the right or to the left of the *x*-axis. This procedure must be performed so that the value of the input variable represents the address of the memory cell where the value of the membership function of the respective qualifier is located. To implement the inference engine model with reconfigurable architecture, several of the classic solutions were analyzed and then the most suitable ones were used to solve the problem in question. The concept of the generic inference engine is required to be defined in the case of solving specific problems of automatic decision-making, algorithms that can change over time. Usually, these systems are characterized by the ability to self-organize the decision-making process, which makes it difficult to design such a system and implement such a decision-making algorithm. The architectures proposed for solving these problems can be used both for the implementation of generalized decision-making algorithms and for ensuring the possibility of dynamic reconfiguration. This methodology is used to describe nonlinear or probabilistic decision-making processes. The use of configurable inference rules in the inference engine structure offers the possibility to change them over time. The use of configurable architectures in the inference engine structure, unlike specialized Fuzzy processors, excludes the redesign of integrated circuits by using FPGA circuits that have many inputs/outputs ports and only require reconfiguration of the circuit. Even the design stage of a new fuzzy kernel can be significantly simplified by using libraries of fuzzy logical elements. The generic inference engine can serve for the development of decision-making algorithms initially implemented in the conditions of insufficient data. Therefore, the use of reconfigurable inference engines offers the possibility to implement different decision-making algorithms in fuzzy systems.

Below, we will mention some Fuzzy Applied Cell Control Technology elements:

⮚ *Declaration type variables*

*Crisp [numeric variable]* = Defines numeric variables belonging to rigid input and output sets, as well as other numeric variables useful in the system

*Subset […]* = Defines variables as fuzzy subsets.

*Varlin [linguistic variable]* = Defines the linguistic variables that will receive fuzzy partitions.

*Term [linguistic term]* = Defines the list of linguistic terms used to describe linguistic variables.

⮚ *Initialization type variables*

*Partition [varlin, term, inf, sup, a, b, c, d]* = Defines the meaning of the linguistic term term associated with the linguistic variable varlin, in the universe of discourse [inf, sup].

*varlin_1 = varlin_2* = Assigns the meanings of the linguistic terms of the variable varlin_1 to the linguistic variable varlin_2. Both variables must be previously defined with the varlin function.

*subfuse as varlin* = The fuzzy subfuse subset is defined relative to the set of linguistic terms of the varlin linguistic variable.

⮚ *Execution variables*

*crisp_1 = crisp_2* = Assigns to numeric variable crisp_1 value of numeric variable crisp_2

*crisp = input (i)* = The numeric variable crisp is assigned the numeric value converted to the analog and processor input

*fuzz* (*crisp)* = The fuzz operator merges the crisp numeric variable defined with the crisp function.

*defuzz* (*crisp)* = The defuzz operator defuses the crisp numeric variable defined with the crisp function.

*output* (*crisp)* = The defusification result is transmitted to the analog output associated with the crisp numeric variable defined with the crisp function.

*if subfuz_1 is term_1 and subfuz_2 is term_2 and… then subfuz_k is term k [(height)]* = Defines inference rules of type if then, where *subfuz_i* are the fuzzy subsets defined with the subset function, and *term_i* are the linguistic terms associated with the fuzzy subset defined relatively to a linguistic variable.

*recv (adr, name, key)* = Initiates the reception of the contents of the name variable from the cell with the address adr.

*send (adr, name, key)* = Initiates the issuance of the contents of the name variable to the cell with the address *adr*, associating the key with it.

## 4. Results

In this chapter, we will exemplify two specific examples: evacuation dynamics using fuzzy control for escape panic flow control and complex decision system of an autonomous vehicle. To implement these solutions, we performed the simulation of algorithms in MATLAB version R2015 and LabVIEW version 2015 to validate the operation and performance. Subsequently, the developed algorithms will be implemented hardware using Fuzzy Applied Cell Control Technology soft computing.

### 4.1. Agent-Based Modeling and Fuzzy Logic for Simulating Pedestrian Crowds in Panic Decision-Making Situations

The increasing occurrence of moments of panic during mass events motivated the study of the impact of panic on crowd dynamics and the simulation of pedestrian flows in panic situations. The lack of understanding of moments of panic continues to cause hundreds of casualties each year, not to mention methodological studies of panic behavior capable of predicting crowd dynamics. Under these conditions, there are thousands of casualties and twice as many accidents caused by the crowd around the world, despite efforts to control the crowd and many security forces. Pedestrian crowd dynamics are generally predictable in high-density crowds, where pedestrians cannot move freely, self-propelled interactions develop between pedestrians. Although each pedestrian has personal preferences, the dynamics of the movement can be shaped as a social force of the crowd. The corresponding forces can be controlled for each individual and represent a different variety of behaviors that can be associated with panic situations, such as avoiding danger, crowding, and pushing [38,39].

**Figure 9:**
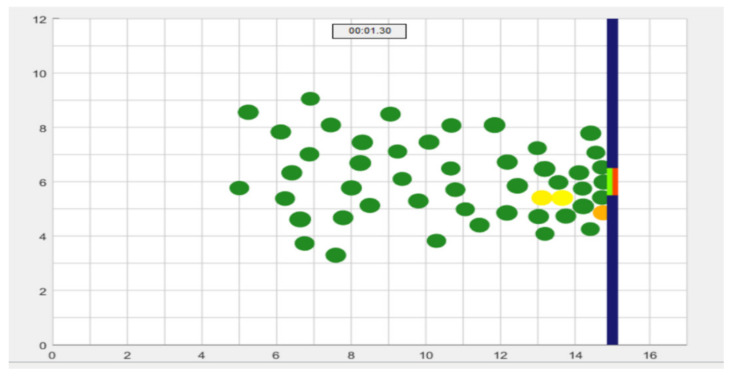
Graphical interface for simulating pedestrian crowds in panic decision-making situations.

In this application we propose a new approach to intelligent control of mobility of people in panic situations for their evacuation to improve the performance of evacuation mobility from closed/open spaces, useful application in operations to save lives, in crisis situations, earthquakes, fires or terrorist acts-CBRNE. In this software development (Figure 9), we use an agent-based model for pedestrian behavior in panic situations to predict collective human behavior in a dynamic crowd situation using fuzzy logic controller. The proposed simulations are a practical way to reduce fatalities and minimize evacuation time (traffic flow) in panic situations.

The application (Figure 9) realizes the optimization of multimer flows, in the case of disasters at events with high population densities in the open air and in closed spaces. Using this application, we follow the execution of evacuation scenarios by decreasing the population density to make efficient evacuations.

Integrated Intelligent Systems. Fusion architectures are the first form of integrated intelligent systems. These include systems that combine different techniques into a single computational model and share data structures and knowledge representations. Another approach is to place different techniques side by side and analyze the interactions between them in a problem-solving task. This method allows the integration of alternative techniques and their simultaneous exploitation. Moreover, the conceptual perspective of the intelligent agent with cognitive ability allows the abstraction of individual techniques and focus on the global behavior of the system but also the study of the individual contribution of each component. The benefits of integrated models include robustness, improved performance and increased troubleshooting capabilities. Finally, fully integrated models provide a number of capabilities such as adaptation, generalization, noise tolerance and reasoning. Merged systems have limitations caused by the increasing complexity of interactions between modules, and specifying, designing and building fully integrated models is an extremely complex process.

**Figure 10:**
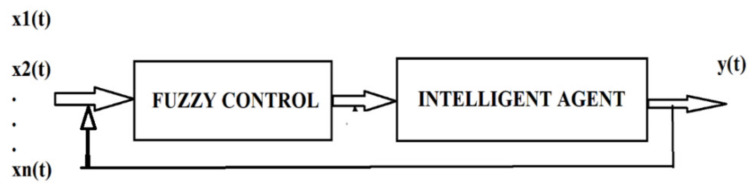
Serial processing architecture made by the fuzzy system and intelligent agents with cognitive ability.

This application is based on serial processing performed by the fuzzy system and intelligent agents, as shown in Figure 10. Before intelligent agents are modeled to determine crowd behavior, the information entered is processed by fusing, and the usefulness of the fuzzy system in this case is when we are dealing with uncertain information before learning intelligent agents takes place. This structure is suitable for the design of systems for determining population dynamics in crisis situations (panic) but also for the control of autonomous robots, as the data collected from unknown and dynamic environments are generally safe. It is necessary to process “fuzzy” data through fuzzy logic. In this way, we can build a perfect model for intelligent agents with cognitive ability through a neural network.

The mathematical models used are multi-agent Artificial Intelligence algorithms, discretization in time and space, realization of autonomous cell type models using fuzzy logic, mathematical modeling of each agent to act individually; the simulation is performed on static tactical field with probabilistic dynamics.

The input data comes from BT detection sensors, which power the RSSI signal reception power and the MAC address of mobile devices, and the output values are distance, crowd dynamics, time horizon and speed. The output results return to the feedback curve in the fuzzification block. The deffuzzification block is based on the centroid method.

To build the sensor system, we used Bluetooth (BT) sensors to receive signals from mobile devices and then used fuzzy logic to determine a person’s location. The goal is to use two fuzzy subsets to characterize the mobile device detected by position and orientation relative to an orientation direction (escape route) [40].

Bluetooth sensors use signal strength (RSSI) and fuzzy logic to estimate the user’s distance from each of the two transmitters installed. The method we use is a pseudo-trilateral that employs fuzzy logic to determine position and orientation based on the power of the receiving signal (RSSI), the MAC address and the actual physical coordinates of the installed Bluetooth access points. Based on the RSSI received by the Bluetooth sensor from the mobile terminal, the distance between the access points and the mobile terminal can be determined.

To streamline the evacuation, we need a pedestrian control system that can automatically optimize the waiting time for each evacuation route based on the number of pedestrians (social crowds) already near the escape route and the number of those who will arrive at the escape route.

Before implementing the application in the Fuzzy Applied Cell Control Technology soft computing language, the solution was simulated using the MATLAB R2015 program.

*Algorithm Design*

Input sizes are:

- Number of pedestrians already on the QUEUE escape route;
- Number of pedestrians who will arrive ARRIVAL.

The output size is

- The evacuation time interval for each pedestrian passing through the EXTENSION escape route.

Value ranges of input and output quantities

Crisp (ARRIVAL) [0 6];

Crisp (QUEUE) [0 6];

Crisp (EXTENSION) [0 12].

Linguistic variables and membership functions

**Figure 11:**
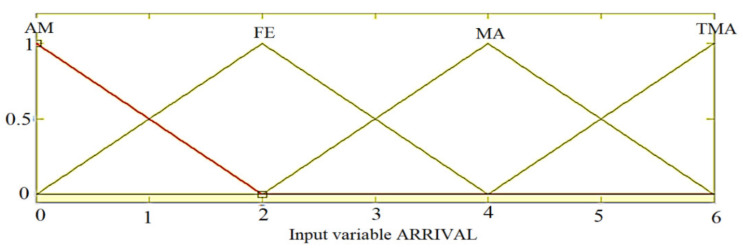
The membership functions of the ARRIVAL entry.

**Figure 12:**
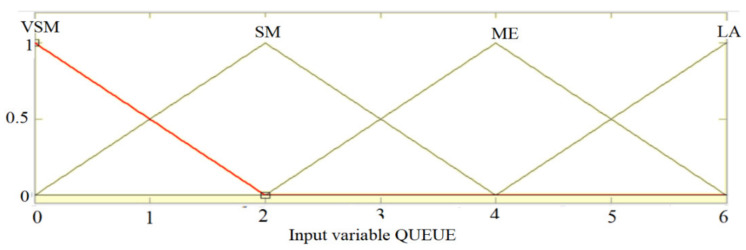
Membership functions of the QUEUE entry.

**Figure 13:**
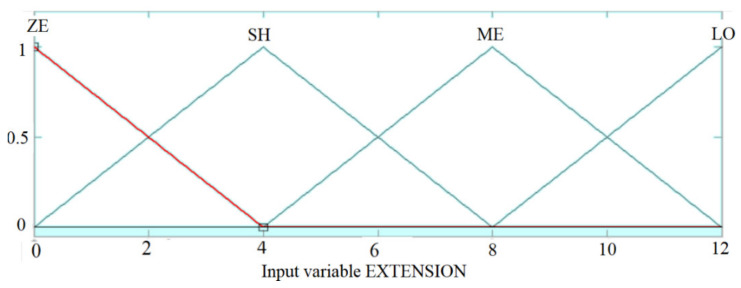
EXTENSION output membership functions.

For the linguistic variables of the regulator the following linguistic terms are chosen:

- For ARRIVAL: ALMOST (AM), FEW (FE), MANY (MA), TOO MANY (TMA);
- For QUEUE: VERY SMALL (VSM), SMALL (SM), MEDIUM (ME), LARGE (LA);
- For EXTENSION: ZERO (ZE), SHORT (SH), MEDIUM (ME), LONGER (LO);

The membership functions are chosen according to the Figure 11, Figure 12 and Figure 13:

The basis of rules are

- If ARRIVAL is AL and QUEUE is VSM, then EXTENSION is ZE;
- If ARRIVAL is AL and QUEUE is SM, then EXTENSION is ZE;
- If ARRIVAL is AL and QUEUE is ME, then EXTENSION is ZE;
- If ARRIVAL is AL and QUEUE is LA, then EXTENSION is ZE;
- If ARRIVAL is FE and QUEUE is VSM, then EXTENSION is SH;
- If ARRIVAL is FE and QUEUE is SM, then EXTENSION is SH;
- If ARRIVAL is FE and QUEUE is ME, then EXTENSION is ZE;
- If ARRIVAL is FE and QUEUE is LA, then EXTENSION is ZE;
- If ARRIVAL is MA and QUEUE is VSM, then EXTENSION is ME;
- If ARRIVAL is MA and QUEUE is SM, then EXTENSION is ME;
- If ARRIVAL is MA and QUEUE is ME, then EXTENSION is SH;
- If ARRIVAL is MA and QUEUE is LA, then EXTENSION is ZE;
- If ARRIVAL is TMA and QUEUE is VSM, then EXTENSION is LO;
- If ARRIVAL is TMA and QUEUE is SM, then EXTENSION is ME;
- If ARRIVAL is TMA and QUEUE is ME, then EXTENSION is ME;
- If ARRIVAL is TMA and QUEUE is LA, then EXTENSION is SH.

Table 1 presents the basis of rules identified for the proposed application.

**Figure 14:**
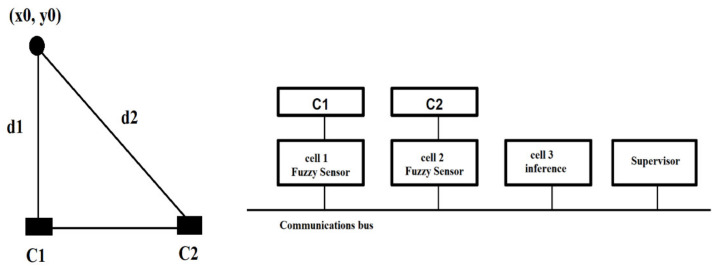
Description of the position of an object, based on two distance measuring sensors.

*Implementing the application in the Fuzzy Applied Cell Control Technology soft computing language.* The problem is to obtain a symbolic description of the position of an object, based on two sensors for measuring distance, *c*_1_ and *c*_2_, represented in Figure 14. The purpose of this application is to characterize the detected object by determining its position and orientation by analyzing two fuzzy subsets. The syntax sets of terms are *{close, quite_close, quite_far, far}* and *{left, front, right}.*

To obtain the distances *d*_1_ and *d*_2_, we use two BT sensors. The system can be extended by introducing the 3rd BT sensor, thus forming pairs of 2 BT sensors each to reduce the error of estimating the position/orientation of an object.

**Figure 15:**
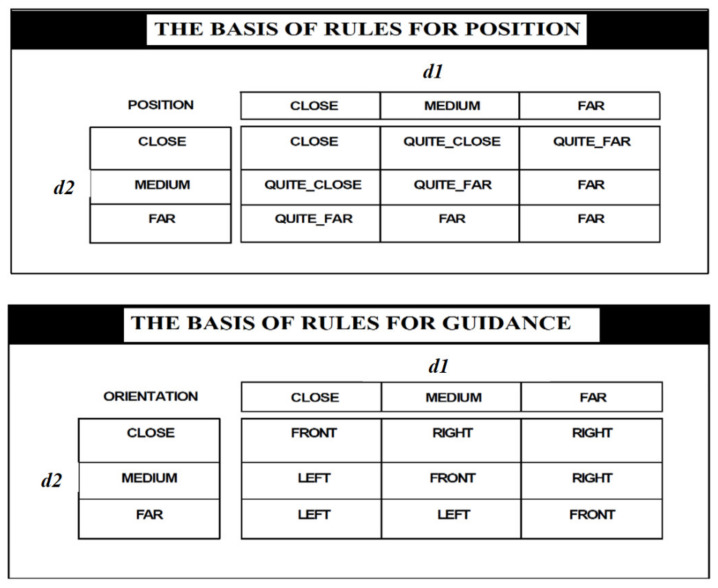
The aggregation is performed by two different sets of rules.

**Figure 16:**
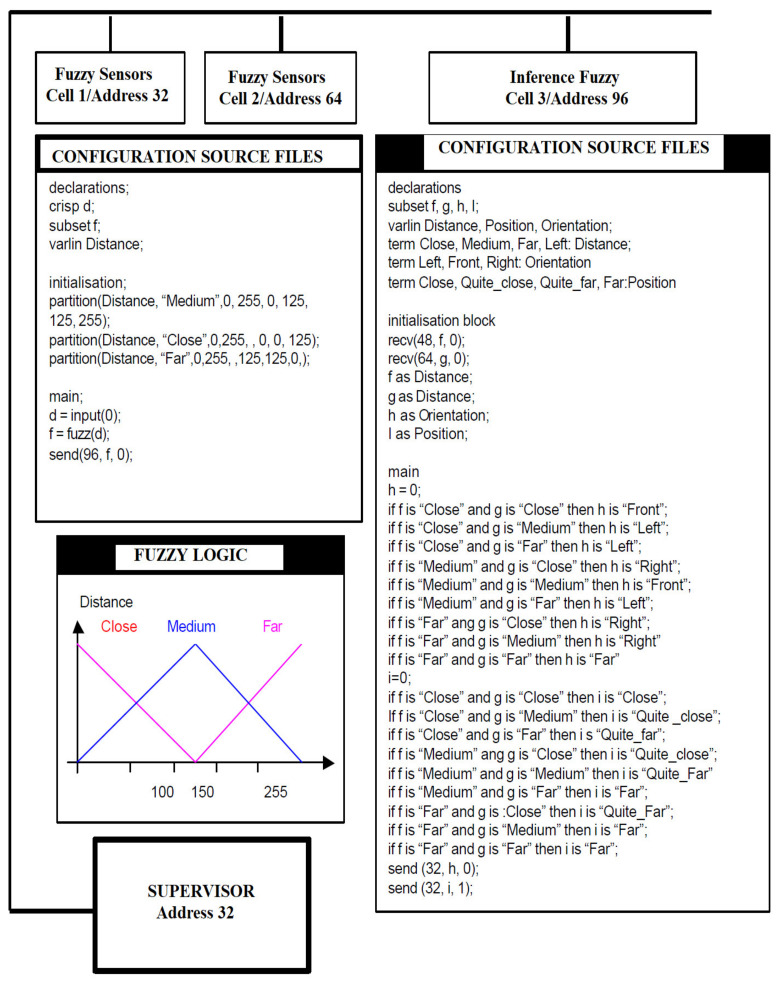
Configuration of fuzzy cells system.

Smart sensors can be turned into fuzzy sensors by simply applying the FACCT programming language, without using additional hardware. Each fuzzy distance sensor describes the measurement as a fuzzy subset over the recognition set {close, medium, headlight}. A third cell is used to aggregate the two distances, which involves creating a virtual sensor to detect obstacles. The aggregation is performed by two different sets of rules (see Figure 15), the first rule being associated with the position and the second rule being associated with the orientation of the objects. The configuration of the fuzzy cells is described in Figure 16. The two configurations are based on the same text files made in the FACCT language. Each cell first performs the fuzzy symbolic transformation of the measurements. The resulting fuzzy subsets are transmitted to cell 3.

For example, suppose that *d*_1_ *=* 90 m and *d*_2_ *=* 230 m. According to the syntax definition of the varying distance shown in Figure 16, the specific fusions are

(8) $$\varphi_{2}\left(d1\right)=\left\{\frac{0.3}{close},\frac{0.7}{mediu},\frac{0}{far}\right\}$$

(9) $$\varphi_{2}\left(d2\right)=\left\{\frac{0}{close},\frac{0.2}{mediu},\frac{0.8}{far}\right\}$$

The proposed operators give for *h* and *i*

(10) $$h=\left\{\frac{0.86}{left},\frac{0.14}{front},\frac{0}{right}\right\}$$

(11) $$i=\left\{\frac{0}{close},\frac{0.06}{quite\_close},\frac{0.38}{quite\_far},\frac{0.56}{far}\right\}$$

**Figure 17:**
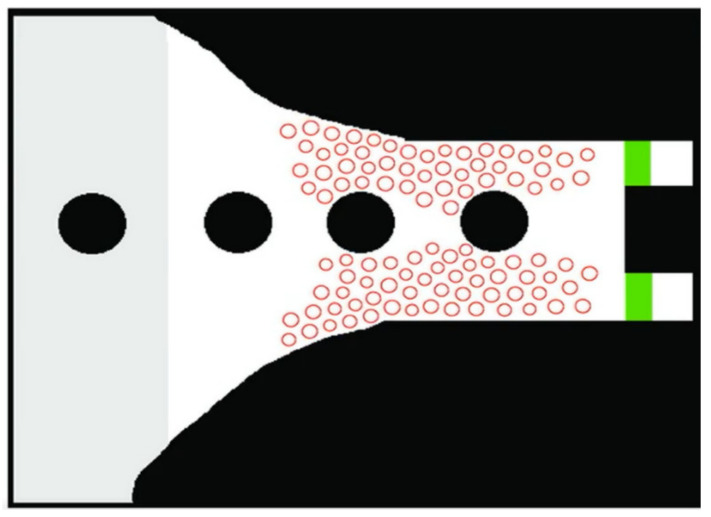
The result of modeling with intelligent cognitive agents.

When designing a system that facilitates the evacuation of a crowd in a panic situation (Figure 17), we must synchronize all consecutive exits (evacuation direction) so that the evacuation of the crowd is as efficient as possible, based on distance and position calculations made by logic fuzzy and based on intelligent agents with cognitive behavior that will determine population dynamics.

### 4.2. Fuzzy Controller for Mobile Robot

In designing robots, they are intended to act more like human beings than machines. Traditional logic makes use of firm numerical values and is therefore not suitable for approximating the human decision-making process. Fuzzy logic mimics the human thought process by using the entire interval between zero and one, being used to represent human thinking quite accurately [41]. The design of a fuzzy logic system can generally be divided into the following stages: fuzzification, inference and defuzzification.

This application demonstrates the use of fuzzy logic to simulate parking a robot in a specified location.

**Figure 18:**
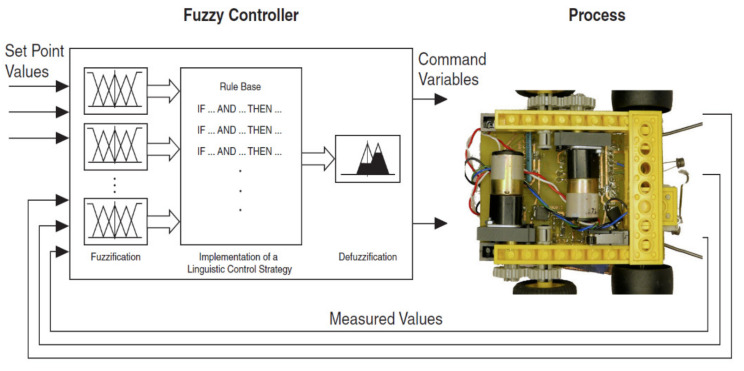
Closed-Loop Control structure with Fuzzy Controller.

**Figure 19:**
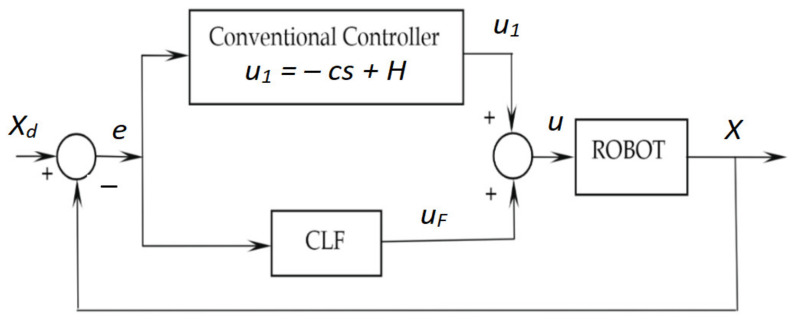
Control system of the robot.

**Figure 20:**
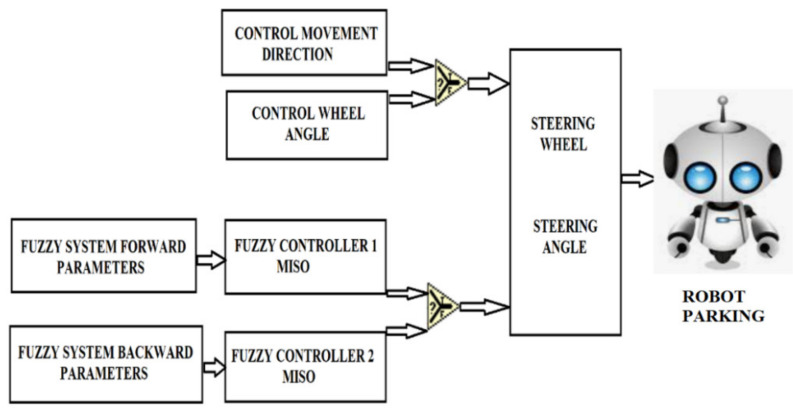
Representation of the architecture for the realization of the route optimization algorithm.

The autonomous robots are an independent mobile system equipped with various mechatronic devices that act on the steering, acceleration and brake, respectively, to travel without the intervention of the driver, in a real environment [41,42,43]. The main functions of an Automobile as Mobile Robot vehicle: perception, recognition and identification of the environment through the external sensory system; location, identifies the spatial position in relation to fixed, sometimes mobile objects in the moving space; mapping, modeling the adjacent environment through maps; planning, generating routes and trajectories; autonomous management, control and command of the execution subsystems for tracking the trajectory requirements; manual guidance, by preserving the primary guiding function by a human leader. Figure 18 and Figure 19 show the system implementation architecture using fuzzy logic and Bezier curves to achieve a route optimization algorithm. Figure 20 illustrates the diagram of the Mamdani fuzzy controller for controlling the mobile robot considered in the present paper.

**Figure 21:**
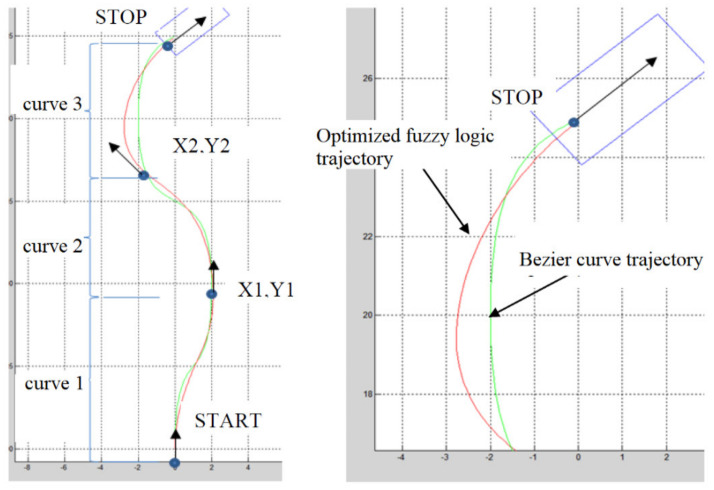
Example for calculation the optimization of the trajectory with Bezier curves and fuzzy.

The algorithm for generating routes based on Bezier curves is based on the idea of fixing an intermediate point and calculating the optimal trajectory that joins the current position of the Mobile Robot with this point. The midpoint is defined by the partition (*varlin,term,inf,sup,a,b,c,d)* instruction, which has as a variable parameter the distance measured from the reference point of the vehicle to the target point to which it is desired to travel locally. The steering angle can be calculated based on this data. To generate the trajectory, it is necessary and sufficient to define the following parameters (Figure 21): START point with position (X0, Y0), speed Gs, curvature Cs, speed Vs, STOP point with position (X4, Y4), speed Gf, curvature Cf and velocity Vf. In addition, intermediate points must be defined, like START/STOP points. This is done by using the data received from the fuzzy algorithm. The importance of trajectory estimation using the extended fuzzy logic, Kalman filter algorithm and Voronoi diagrams has been detailed in articles [44,45], in this article using Beziers curves.

Thus, when a jump in the path is detected that exceeds a previously established value, an intermediate point (Xi, Yi) is placed. This intermediate point is then used as a control point for generating the Bezier curve.

**Figure 22:**
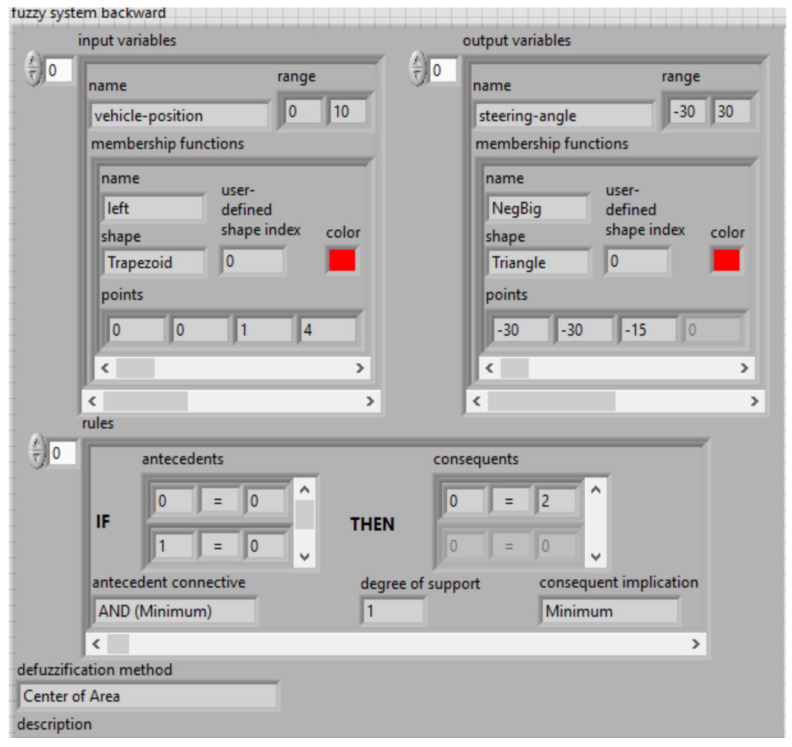
LabVIEW implementation of the fuzzy controller system for backward trajectory optimization.

**Figure 23:**
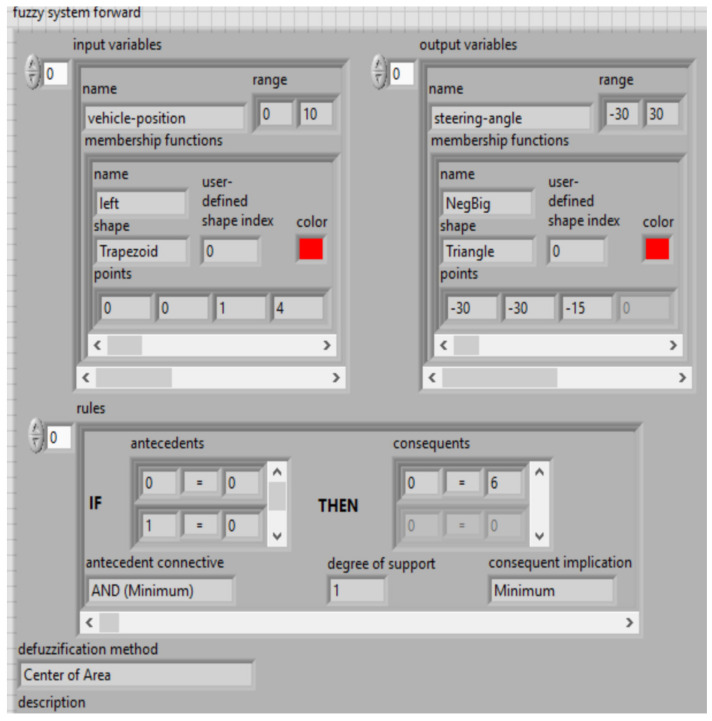
LabVIEW implementation of the fuzzy controller system for forward trajectory optimization.

Fuzzy functions of expert knowledge encapsulated within statistical workflow data are shown in Figure 22 and Figure 23, simulate software in LabVIEW version 2015.

Dual inputs/outputs Fuzzy Controller (Figure 19) that is used to automate the maneuvering process leading a truck from an arbitrary start position in backward direction to a loading ramp. The robot is supposed to be run at constant low speed. The maneuvering algorithm is represented by an appropriate rule base (knowledge basis). The current maneuvering situation is at least represented by the two linguistic input variables “vehicle-position” towards the loading ramp position and “vehicle-orientation”. The controller output variable “steering-angle” serves as process command variable. This Fuzzy Controller is responsible for the normal backward manneuvering operations.

**Figure 24:**
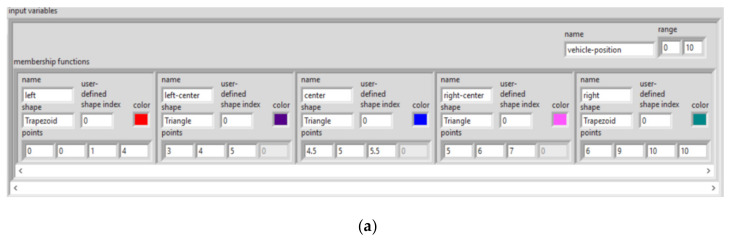

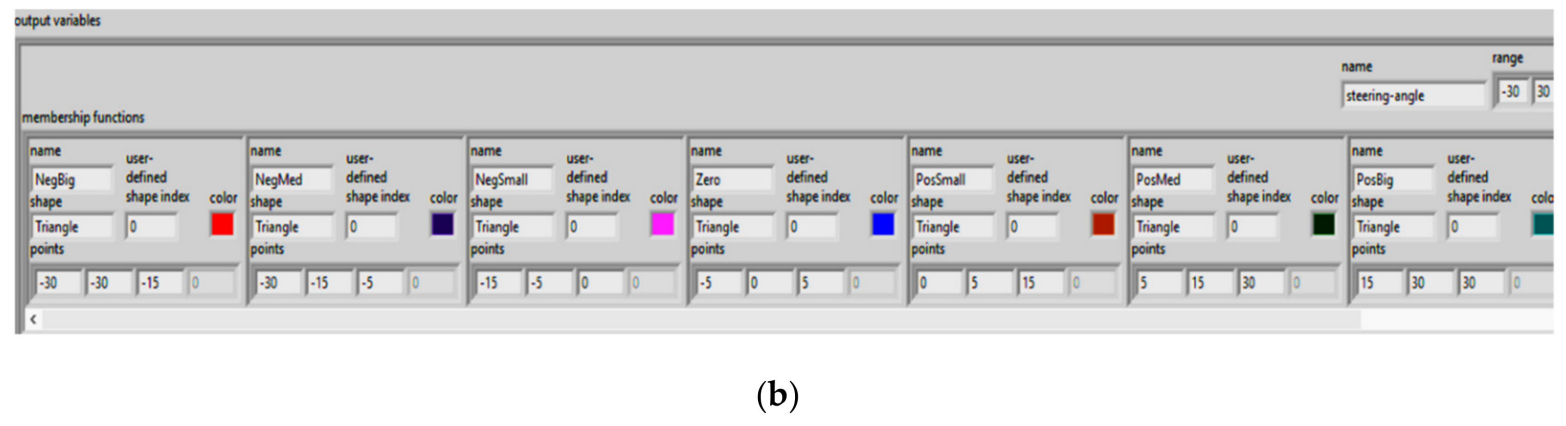
LabVIEW implementation of the basis of rules for input variables (**a**) and output variables (**b**)—membership functions.

Dual input Fuzzy Controller (Figure 20) is used to automate the maneuvering process leading a truck from an arbitrary start position in backward direction to a loading ramp. The robot is supposed to be run at constant low speed. The maneuvering algorithm is represented by an appropriate rule base (knowledge basis). The current maneuvering situation is at least represented by the two linguistic input variables “vehicle-position” towards the loading ramp position and “vehicle-orientation”. The controller output variable “steering-angle” serves as process command variable. This Fuzzy Controller is responsible for foreward maneuvering operations necessary to bring the vehicle in a good start position when backward operation fails to hit the ramp. The basis of rules is shown in Figure 24.

**Figure 25:**
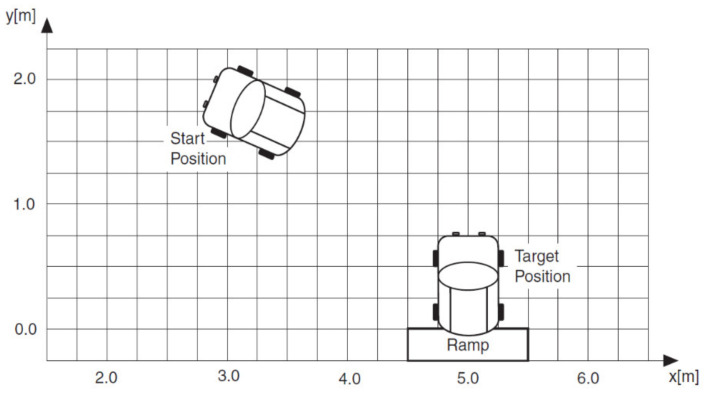
Automating robot parking.

Consider an example in which you want to automate a robot to park itself from an arbitrary starting position. A driver can control the vehicle by constantly evaluating the current status of the vehicle, such as the distance from the target position and the orientation of the vehicle, to derive the correct steering angle. Figure 25 represent this example.

You can define two input linguistic variables for this example. Vehicle Position *x* represents the vehicle position in relation to the destination. Vehicle Orientation β represents the orientation of the vehicle. You also can define an output linguistic variable, Steering Angle ϕ, to represent the steering angle of the vehicle that you want to control. You can define linguistic terms of Left, Left Center, Center, Right Center and Right for the Vehicle Position *x* input linguistic variable to describe the possible positions of the vehicle in relation to the destination. You can define linguistic terms of Left Down, Left, Left Up, Up, Right Up, Right and Right Down for the Vehicle Orientation β input linguistic variable to describe the possible orientations of the vehicle. The linguistic terms of the Steering Angle ϕ output linguistic variable must represent both the direction and magnitude that the steering angle changes. Therefore, you can use the linguistic terms Negative Large, Negative Medium, Negative Small, Zero, Positive Small, Positive Medium and Positive Large for this output linguistic variable.

Membership functions are numerical functions corresponding to linguistic terms. A membership function represents the degree of membership of linguistic variables within their linguistic terms. For example, the linguistic variable Vehicle Position *x* might have full membership (1) within the linguistic term *Center* at 5 m, no membership (0) within that term at 4 m or less and 6 m or greater and partial membership at all distances between 4 and 6 m. If you plot the degree of membership against the value of Vehicle Position *x*, you can see that the resulting membership function is a triangle function.

**Figure 26:**
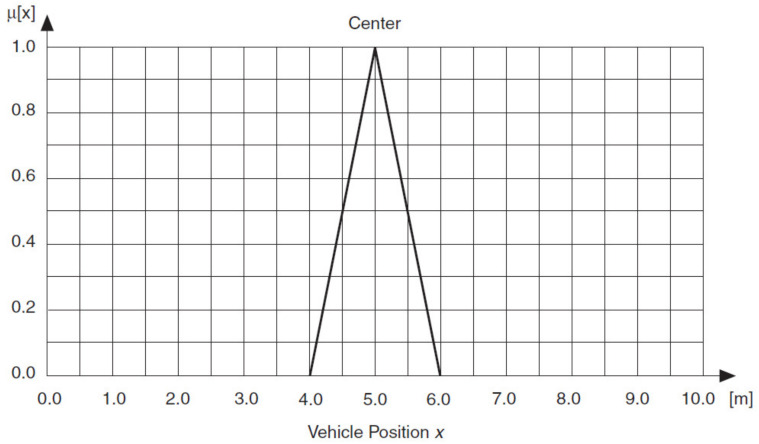
Triangular membership function for the linguistic term *Center.*

**Figure 27:**
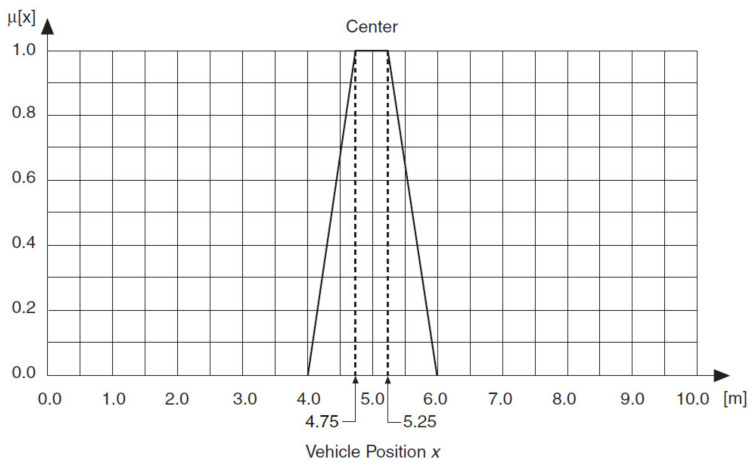
Trapezoidal membership function for the linguistic term *Center.*

Sometimes a linguistic variable has full membership within a linguistic term at a range of values rather than at a point value. If, for example, the linguistic variable Vehicle Position *x* has full membership within the linguistic term *Center* at values *x* = 5 ± 0.25 m, a trapezoidal membership function applies, as shown in Figure 26 and Figure 27.

**Figure 28:**
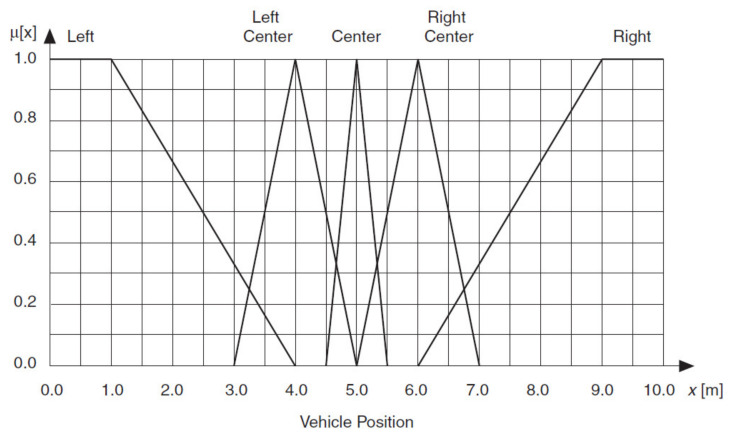
Membership functions for Robot Position *x.*

**Figure 29:**
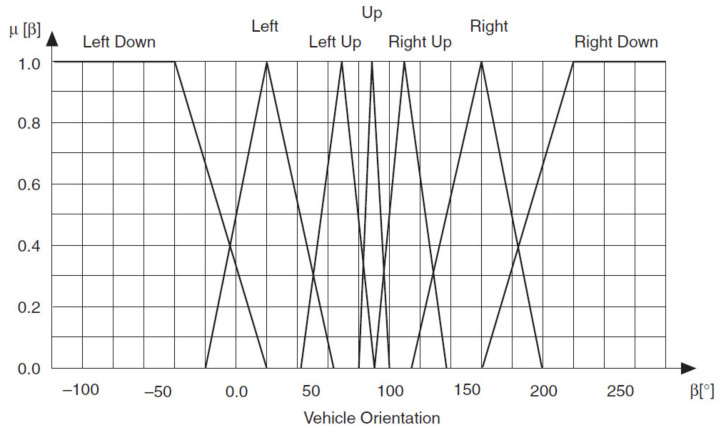
Membership functions for Robot Orientation β.

**Figure 30:**
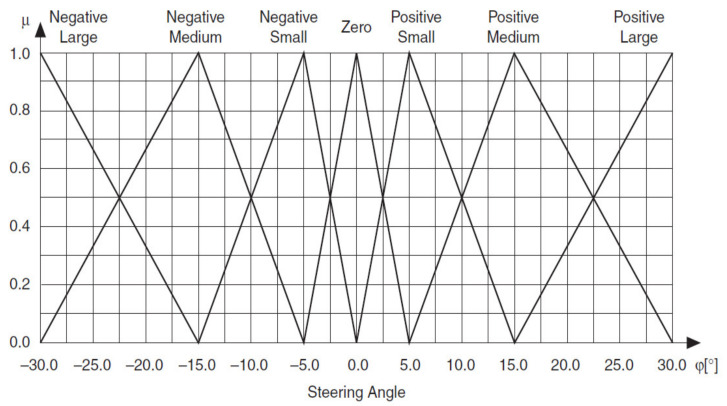
Membership functions for Steering Angle φ.

Figure 28, Figure 29 and Figure 30 show all membership functions for the input and output linguistic variables of the robot maneuvering fuzzy system.

Rules describe, in words, the relationships between input and output linguistic variables based on their linguistic terms. A rule base is the set of rules for a fuzzy system.

To create a rule, you must specify the antecedents, or IF portions, and consequents, or THEN portions, of the rule. For example, consider the following rule: IF Robot Position *x* is *Left Center* AND Robot Orientation β is *Left Up*, THEN Steering Angle φ is Positive Small. The clauses “*Robot Position x* is *Left Center*” and “*Robot Orientation* β is *Left Up*” are the antecedents of this rule. The clause “*Steering Angle* ϕ is *Positive Small*” is the consequent of this rule.

Associate an input linguistic variable with a corresponding linguistic term to form an antecedent. Associate an output linguistic variable with a corresponding linguistic term to form a consequent. The consequent of a rule represents the action you want the fuzzy controller to take if the linguistic terms of the input linguistic variables in the rule are met. When constructing a rule base, avoid contradictory rules, or rules with the same IF portion but different THEN portions. A consistent rule base is a rule base that has no contradictory rules.

The total number *N* of possible rules for a fuzzy system is defined by the following equation:

$$N=p_{1}\times p_{2}\times \ldots \ldots .\times p_{n}$$

where *pn* is the number of linguistic terms for the input linguistic variable *n*. If each input linguistic variable has the same number of linguistic terms, the total number *N* of possible rules is defined by the following equation:

$$N=p^{m}$$

where *p* is the number of linguistic terms for each input linguistic variable, and *m* is the number of input linguistic variables. For example, for three input linguistic variables with five linguistic terms each, the total number of possible rules is $N=5^{3}=125$.

A rule base with at least one active rule for each possible combination of input linguistic variables and linguistic terms is a complete rule base. If you define an incomplete rule base, you must specify a default linguistic term for each output linguistic variable so the fuzzy controller can handle situations in which no rules are active.

**Figure 31:**
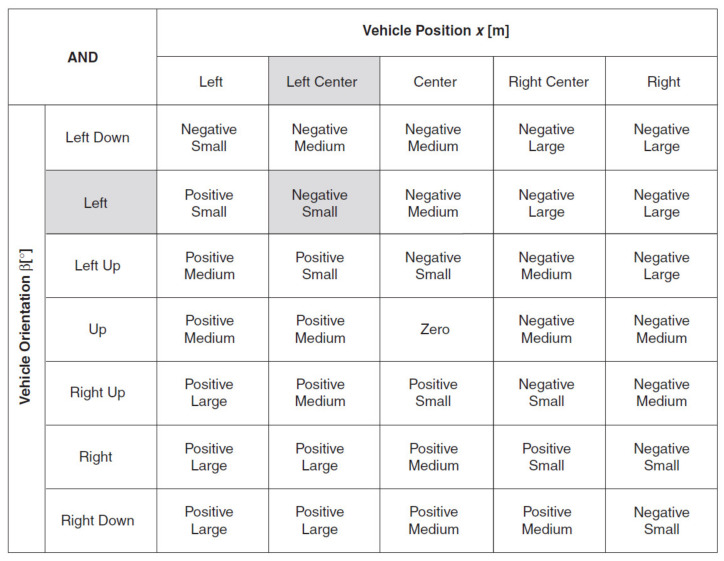
Complete rule base for the robot maneuvering.

The *Robot Position x* input linguistic variable has five linguistic terms, and the *Robot Orientation* β linguistic variable has seven linguistic terms. Therefore, the rule base of the vehicle maneuvering example consists of N=5×7=35 rules. You can document the complete rule base in matrix form, as shown in Figure 31.

Each column or row represents an antecedent of a rule. The term at the intersection of a column and a row is the consequent of the rule corresponding to the aggregated rule antecedent. For example, the following rule is highlighted in Figure 31. IF *Robot Position x* is *Left Center,* AND *Robot Orientation* β is *Left*, THEN *Steering Angle* φ is *Negative Small*.

The fuzzy logic controller then uses the following equation to calculate the geometric center of this area.

$$CoA=\frac{\int_{x_{min}}^{x_{max}}f\left(x\right)xdx}{\int_{x_{min}}^{x_{max}}f\left(x\right)dx}\;$$

where CoA is the center of area, *x* is the value of the linguistic variable, and *x_min_* and *x_max_* represent the range of the linguistic variable. The Center of Area defuzzification method effectively calculates the best compromise between multiple output linguistic terms.

**Figure 32:**
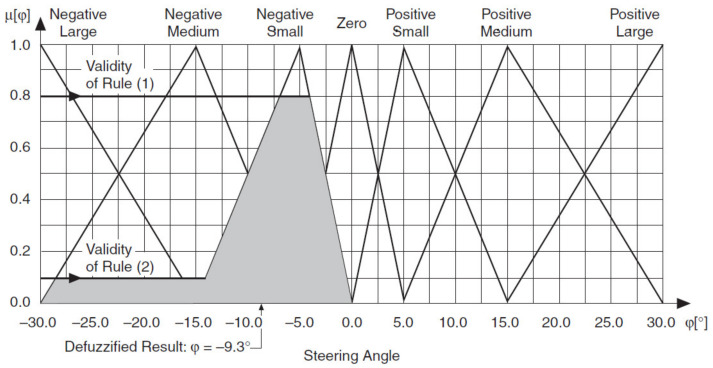
Center of Area (CoA) Defuzzification Method.

Figure 32 illustrates the Center of Area (CoA) defuzzification method for the *Steering Angle* ϕ output linguistic variable, assuming the minimum implication method. The shaded portion of the graph represents the area under the scaled membership functions.

**Figure 33:**
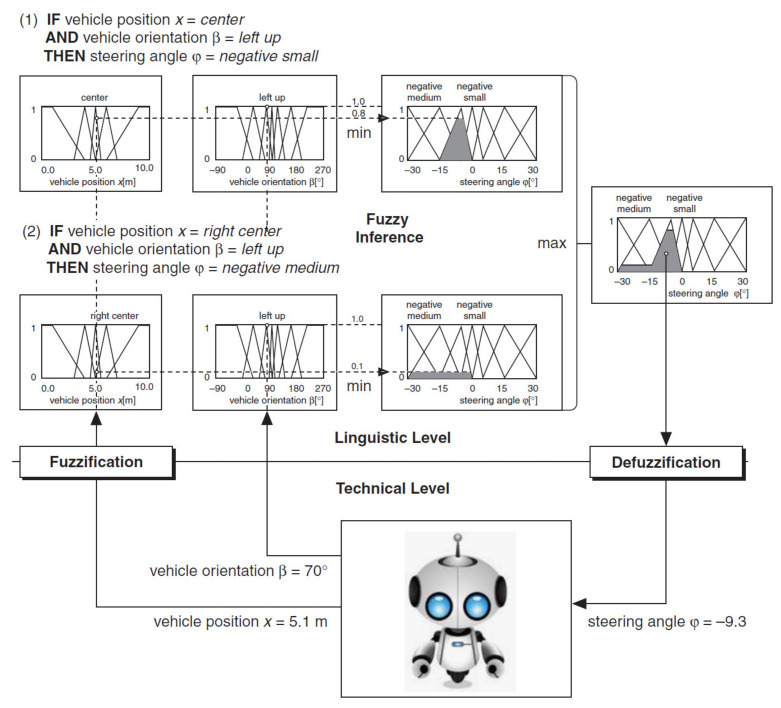
Process of a Fuzzy Controller using CoA Defuzzification.

Figure 33 summarizes the process of a fuzzy controller for the robot maneuvering, using the CoA method of defuzzification.

## 5. Discussion

After explaining the concept of distributed intelligent control, this article described the concept of fuzzy components and their definition in terms of hardware and software. A simple solution to implement fuzzy cell-based systems has been described. The cells configuration in the network is performed by a specific syntax, called Fuzzy Applied Cell Control Technology. The advantages of using a network of fuzzy components does not limit only to those offered by the distributed systems. We find that fuzzy cells are easy to configure, offering high-level programming functions, such as mergers and decision-making processes. The distributed network configuration allows the addition of fuzzy cells to increase system performance. The added components are not necessarily necessary to be fuzzy components, because the network supports a multitude of data, thus creating an IoT network. There is also the possibility to create virtual components, where cell 3 represents a virtual position sensor.

The main contributions that the research presented in the article makes are the following:

⮚ Defining the concept of distributed fuzzy control.
⮚ Defining fuzzy components in a system with distributed fuzzy control.
⮚ Defining the operations of symbolic fusing, symbolic inference and fuzzy–real symbolic transformation based on the notions of fuzzy meaning and fuzzy description.
⮚ Defining the elements of an interoperable language Fuzzy Applied Cell Control Technology for the development of fuzzy components with distributed intelligence.

Within the theoretical and experimental research carried out, especially for application *Fuzzy controller for mobile robot*, two essential problems were solved: the mapping of the environment to be traveled and the planning of the Automobile as Mobile Robot trajectory. From the point of view of mapping, efficient methods and algorithms have been proposed from the point of view of calculation, able to consider uncertainties, to accurately model the operating environments as well as to navigate autonomously according to the optimal trajectories. In addition, issues related to simultaneous localization and mapping issues related to the extraction of features and the issue of association have been resolved. Obtaining credible information from sensors as well as the accuracy of identifying the previous exploration of the same territory are key elements for the convergence of a simultaneous localization and mapping algorithm.

We implemented a baking calculation of the route using Bezier curves. To simulate the closed loop system, the fuzzy logic controller has been implemented for automatic backward parking control. The algorithm is based on the initial position of the robot and the final position, and the logical Fuzzy controller will control the steering angle and speed of the robot, so that the robot maintains its course towards the parking position. The parking error was reduced (minimized) by designing the fuzzy inference system in the fuzzy logic controller. However, the results obtained from the simulation showed an orientation accuracy of 94%. We will continue research to modify the fuzzy rules algorithm to improve back parking accuracy.

The navigation algorithms for planning the designed, implemented and tested navigation routes meet the requirements imposed by the total autonomy, being able to model, generate and follow in real time the complicated trajectories to avoid obstacles that appear randomly in the operating environment. The validation of the algorithms designed, developed and implemented was done based on simulations, followed by laboratory experiments.

## 6. Conclusions

From the point of view of the concept of distributed intelligent control, three types of components can be defined: Fuzzy sensors, which provide a representation of measurements as fuzzy subsets; Fuzzy actuators, which can act in a real world; depending on the fuzzy subsets; and they also receive fuzzy components of inference, which can perform distributed fuzzy logic. They produce new fuzzy subsets from the fuzzy subsets they received. Fuzzy components can be integrated for different applications into a compact model of components, called the fuzzy cell. The configuration of the cells in the network is done through a specific programming language.

The advantages of using a network of fuzzy components are not only those offered by distributed systems, because fuzzy cells are easy to configure, offering high-level functions such as mergers and decision-making processes. The presented application Agent-based modeling and fuzzy logic for simulating pedestrian crowds in panic decision-making is based on the implementation of a network of sensors for receiving Bluetooth signals from mobile devices and the use of fuzzy logic to determine the position of pedestrians towards escape routes and calculation of the optimal evacuation distance. The purpose of this research was to develop a solution for locating, tracking and analyzing populations in panic situations, with different fields of application, from public transport to Crisis Management systems. The accuracy of locating Bluetooth targets in indoor environments depends on the efficiency of the data processing algorithms. The activities presented in this paper focused mainly on the implementation of distance calculus using fuzzy logic to improve the tracking of current positioning algorithms (trilateral and triangular). An improved BT positioning algorithm is proposed through the merger using fuzzy control and intelligent multi-agent algorithms. We created a new intelligent control interface for evacuation of people in panic situations in order to improve the performance of evacuation mobility from closed/open spaces, a useful application in operations to save lives in crisis situations, earthquakes, fires or terrorist actions-CBRNE.

We have successfully designed a fuzzy controller that solves the problem of navigating a robot in terms of obstacle avoidance behaviors, wall tracking, exploration and tracking of the predetermined trajectory. One of the main contributions is the development of the sensor-based navigation system, a system that uses fuzzy logic. Two types of fuzzy controllers were developed, and simulation demonstrated that the fuzzy controller based on the Bezier function is better suited for the development of robotic navigation systems. The uniqueness of this application comes from the use of fuzzy Mamdani type logic with two outputs. In the revised literature, many authors either avoided the use of the fuzzy two-output logic controller or developed their own application program by using various programming languages. In this application, we use standard fuzzy controller development tools for autonomous mobile robot navigation.

### Future Research Directions

The research carried out as well as the theoretical, experimental and practical results obtained, integrated in a logical structure for obtaining autonomous robotic systems, only partially cover the diversity of issues highlighted after the current stage. From the analysis of the studies carried out as well as of the results obtained within this paper, four main directions of their continuation are highlighted:

Implementation of the results obtained on real complex systems for telecommunications;

Implementation of the results obtained on real systems for navigation in real environments (industrial, road);

Implementation of the traffic flow algorithm in an ITS management system at the level of a city;

Implementation of the traffic flow algorithm in complex telecommunication networks;

Development of virtual reality environments for complex integrated systems used in communication networks;

Development of virtual reality environments for learning Automobile as Mobile Robot systems as well as the development of a high-performance product dedicated to their programming by learning.

Studies on multimodal human-vehicle communication interfaces correlated with the new trends in cognitive programming of robots.

## 7. Patents

Part of this research has been previously tested for developing a method for the anonymous collection of travelers flowing in a public transport system and resulted in a Patent proposal: ROA/00493, “Method and System for Anonymous Collection of Information regarding Position and Mobility in Public Transportation, employing Bluetooth and Artificial Intelligence” in 2019.

## Figures and Tables

**Table 1:** Basis of rules identified for the proposed application.

ARRIVAL	VSM	QUEUE SM	ME	LA
AL	ZE	ZE	ZE	ZE
FEW	SH	SH	ZE	ZE
MA	ME	ME	SH	ZE
TMA	LO	ME	ME	SH

## Data Availability

The data used in this study were obtained in the tests performed in the Artificial Intelligence laboratory within the Faculty of Transport, Polytechnic University of Bucharest, Romania.

## References

[1] Sharma R., Gaur P., Mittal A.P. (2016). Design of two-layered fractional order fuzzy logic controllers applied to robotic manipulator with variable payload. Appl. Soft Comput..

[2] Hu H., Wang X., Chen L. (2020). Impedance with Finite-Time Control Scheme for Robot-Environment Interaction. Math. Probl. Eng..

[3] Saleki A., Mehdi M. (2020). Model-free control of electrically driven robot manipulators using an extended state observer. Comput. Electr. Eng..

[4] Abror B., Hyun K., Myeong C., Rymduck O., Akmal A., Heung S. (2019). Application of fuzzy logic for problem of evaluating states of a computing system. Appl. Sci..

[5] Bhatia V., Kalaichelvi V., Karthikeyan R. Application of a Novel Fuzzy Logic Controller for a 5-DOF Articulated Anthropomorphic Robot. Proceedings of the 2015 IEEE International Conference on Research in Computational Intelligence and Communication Networks (ICRCICN).

[6] Ghanooni P., Yazdanib A.M., Mahmoudi A., MahmoudZadeh S., Ahmadi Movaheded M., Fathi M. (2020). Robust precise trajectory tracking of hybrid stepper motor using adaptive critic-based neuro-fuzzy controller. Comput. Electr. Eng..

[7] Mahmoodabadi M.J., Abedzadeh Maafi R., Taherkhorsandi M. (2017). An optimal adaptive robust PID controller subject to fuzzy rules and sliding modes for MIMO uncertain chaotic systems. Appl. Soft Comput..

[8] Edalati L., Khaki Sedigh A., Aliyari Shooredeli M., Moarefianpour A. (2018). Adaptive fuzzy dynamic surface control of nonlinear systems with input saturation and time-varying output constraints. Mech. Syst. Signal. Process..

[9] Mamdani E.H. (1974). Application of fuzzy algorithms for control of simple dynamic plant. Proc. Inst. Electr. Eng..

[10] Jassbi J.J., Serra P.J.A., Ribeiro R.A., Donati A. A Comparison of Mandani and Sugeno Inference Systems for a Space Fault Detection Application. Proceedings of the 2006 World Automation Congress.

[11] Zhang Y., Wang J., Han D., Wu H., Zhou R. (2017). Fuzzy-logic based distributed energy-efficient clustering algorithm for wireless sensor networks. Sensors.

[12] Yuste-Delgado A.J., Cuevas-Martinez J.C., Triviño-Cabrera A. (2019). EUDFC-Enhanced Unequal Distributed Type-2 Fuzzy Clustering Algorithm. IEEE Sens. J..

[13] Baghli F.Z., Bakkali E.L., Lakhal Y. (2015). Multi-input multi-output fuzzy logic controller for complex system: Application on two-links manipulator. Procedia Technol..

[14] Xu J.X., Guo Z.Q., Lee T.H. (2013). Design and implementation of a Takagi-Sugeno-Type fuzzy logic controller on a two-wheeled mobile robot. IEEE Trans. Ind. Electron..

[15] Huang C.-H., Wang W.-J., Chiu C.-H. (2011). Design and implementation of fuzzy control on a two-wheel inverted pendulum. IEEE Trans. Ind. Electron..

[16] Chih H., Ya F. (2019). Design of Takagi-Sugeno fuzzy control scheme for real word system control. Sustainability.

[17] Su X., Wu Y., Song J., Yuan P. (2018). A Fuzzy Path Selection Strategy for Aircraft Landing on a Carrier. Appl. Sci..

[18] Zavlangas P.G., Tzafestas S.G., Althoefer K. (2000). Fuzzy Obstacle Avoidance and Navigation for Omnidirectional Mobile Robots.

[19] Nadour M., Boumehraz M., Cherroun L., Puig Cayuela V. (2019). Hybrid type-2 fuzzy logic obstacle avoidance system based on horn-schunck method. Electroteh. Electron. Autom..

[20] Jahanshahi H., Jafarzadeh M., Sari N.N., Pham V.T., Huynh V.V., Nguyen X.Q. (2019). Robot motion planning in an unknown environment with danger space. Electronics.

[21] Lin J., Zhou J., Lu M., Wang H., Yi A. (2020). Design of Robust Adaptive Fuzzy Controller for a Class of Single-Input Single Output (SISO) Uncertain Nonlinear System. Math. Probl. Eng..

[22] Rossomando F., Serrano E., Soria C., Scaglia G. (2020). Neural Dynamics Variations Observer Designed for Robot Manipulator Control Using a Novel Saturated Control Technique. Math. Probl. Eng..

[23] Chatterjee A., Watanabe K. (2005). An adaptive fuzzy strategy for motion control of robot manipulators. Soft Comput..

[24] Bandara R.N., Gaspe S. Fuzzy logic controller design for an Unmanned Aerial Vehicle. Proceedings of the IEEE International Conference on Information and Automation for Sustainability.

[25] Prakash M., Jajulwar K. (2016). Design of adaptive fuzzy tracking controller for Autonomous navigation system. Int. J. Recent Trend Eng. Res..

[26] Xue H., Zhang Z., Wu M., Chen P. (2019). Fuzzy Controller for Autonomous Vehicle Based on Rough Sets. IEEE Access.

[27] Karras G.C., Fourlas G.K. (2020). Model Predictive Fault Tolerant Control for Omni-directional Mobile Robots. J. Intell. Robot. Syst..

[28] Siegwart R., Nourbakhsh I.R., Scaramuzza D. (2011). Introduction to Autonomous Mobile Robots.

[29] Mac T., Copot C., De Keyser R., Tran T., Vu T. (2016). MIMO fuzzy control for autonomous mobile robot. J. Autom. Control. Eng..

[30] Bobyr M.V., Kulabukhov S.A., Milostnaya N.A. Fuzzy control system of robot angular attitude. Proceedings of the 2nd International Conference on Industrial Engineering, Applications and Manufacturing.

[31] Li K., Zhao X., Sun S., Tan M. (2018). Robust target tracking and following for a mobile robot. Int. J. Robot. Autom..

[32] Pandey A., Pandey S., Parhi D. (2017). Mobile robot navigation and obstacle avoidance techniques: A review. Int. Robot. Autom. J..

[33] Steels L., Brooks R. (2018). The Artificial Life Route to Artificial Intelligence: Building Embodied, Situated Agents.

[34] Kolbari H., Sadeghnejad S., Parizi A.T., Rashidi S., Baltes J.H. Extended fuzzy logic controller for uncertain teleoperation system. Proceedings of the 4th International Conference on Robotics and Mechatronics.

[35] Benli E., Motai Y., Rogers J. (2019). Human behavior-based target tracking with an omni-directional thermal camera. IEEE Trans. Cogn. Dev. Syst..

[36] Zadeh L. (1996). Fuzzy logic = computing with words. IEEE Trans. Fuzzy Syst..

[37] Herrera F., Herrera-Viedma E. (2000). Linguistic Decision Analysis: Steps for Solving Decision Problems under Linguistic Information. Fuzzy Sets Syst..

[38] Versaci M., Calcagno S., Cacciola M., Morabito F., Palamara I., Pellicanò D., Burrascano P., Callegari S., Montisci A., Ricci M., Versaci M. (2015). Innovative Fuzzy Techniques for Characterizing Defects in Ultrasonic Nondestructive Evaluation. Ultrasonic Nondestructive Evaluation Systems.

[39] Roboaca S., Dumitrescu C., Mantan I. (2020). Aircraft Trajectory Tracking using Radar Equipment with Fuzzy Logic Algorithm. Mathematics.

[40] Helbing D., Farkas S., Vicsek T. (2020). Simulating Dynamic Features of Escape Panic. Nature.

[41] Abdulla A., Maria J., Francisco M., Arturo E., Jose M. (2019). An Appearance-Based Tracking Algorithm for Aerial Search and Rescue Purposes. Sensors.

[42] Minea M., Dumitrescu C., Costea I., Chiva I., Semenescu A. (2020). Developing a Solution for Mobility and Distribution Analysis Based on Bluetooth and Artificial Intelligence. Sensors.

[43] Terano T., Asal K., Sugeno M. (1992). Fuzzy Systems Theory and Its Applications.

[44] Kasabanov N., Kozma R. (1999). Neuro-Fuzzy Techniques for Intelligent Information Systems.

[45] Kahraman C., Kabak O. (2018). Fuzzy Statistical Decisio-Making: Theory and Applications.

